# Fructose intake and its association with relative telomere length: an exploratory study among healthy Lebanese adults

**DOI:** 10.3389/fnut.2023.1270124

**Published:** 2023-10-31

**Authors:** Nairie Messerlian, Nathalie Zgheib, Fatima Al Zahraa Chokor, Mona Nasrallah, Hani Tamim, Lara Nasreddine

**Affiliations:** ^1^Department of Nutrition and Food Sciences, Faculty of Agricultural and Food Sciences, American University of Beirut, Beirut, Lebanon; ^2^Department of Pharmacology and Toxicology, Faculty of Medicine, American University of Beirut, Beirut, Lebanon; ^3^Department of Public Health, College of Health Sciences, Qatar University Health, Qatar University, Doha, Qatar; ^4^Division of Endocrinology, Department of Internal Medicine, Faculty of Medicine, American University of Beirut Medical Center, Beirut, Lebanon; ^5^Vascular Medicine Program, American University of Beirut Medical Center, Beirut, Lebanon; ^6^Department of Internal Medicine, American University of Beirut Medical Center, Beirut, Lebanon; ^7^College of Medicine, Alfaisal University, Riyadh, Saudi Arabia

**Keywords:** fructose, intake, relative telomere length, adults, Lebanon

## Abstract

**Introduction:**

Shorter relative telomere length (RTL) has been associated with increased incidence of morbidity. Although still disputed, available evidence suggests that dietary factors, including sugar-sweetened beverages (SSB) may be linked with shorter RTL. It was argued that the link between SSB and RTL may be explained by the sugar content of these beverages, and specifically fructose given its impact on oxidative stress and the inflammatory response. However, none of the existing studies have examined the specific link between fructose intake and RTL. This exploratory study aimed at (1) assessing the intake of dietary fructose (total, added and natural) in Lebanese healthy adults and (2) examining dietary fructose as a predictor of short telomere length.

**Methods:**

Following a cross-sectional design (*n* = 282), anthropometric and biochemical data were collected. RTL was assessed by utilizing real-time polymerase chain reaction (RT-qPCR) to amplify both telomere and single-copy gene segments. Dietary intake was evaluated using a culture-specific food frequency questionnaire (FFQ). Intakes of added fructose, naturally-occurring fructose, and total fructose were estimated.

**Results:**

Mean intakes of added and natural fructose were of 39.03 ± 34.12 and 12.28 ± 8.59 g/day, respectively, representing 4.80 ± 3.56 and 1.78 ± 1.41% of total energy intake (EI). Mean total fructose intake was of 51.31 ± 35.55 g/day, contributing 6.58 ± 3.71% EI. Higher intakes of total and added fructose were significantly associated with shorter RTL 2nd RTL tertile as compared to the 3rd RTL tertile; relative risk ratio (RRR) = 3.10 [95% confidence interval (CI): 1.38, 6.94] and RRR = 2.33 (95% CI: 1.02, 5.36), respectively after adjustment for confounders identified using a directed acyclic graph (DAG).

**Conclusion:**

In conclusion, although we could not observe a dose-dependent relation between fructose intakes and RTL shortening and although the study is limited by its small sample size, the findings suggest that total and added dietary fructose intakes may be associated with shorter RTL. Larger studies, of longitudinal nature, are needed to further confirm the study findings.

## 1. Introduction

Telomeres play an essential role in preserving the genome’s structural integrity and protecting chromosomes from end-to-end fusion and degradation (1). However, the length of telomeres shortens progressively with age in different cells and tissues (2). Telomere length may also be perceived as a possible biomarker of biological age, reflecting the cumulative load of inflammation and oxidative stress (3, 4). In fact, a growing body of clinical and epidemiological evidence indicates that the accelerated reduction in telomere length is associated with age-related diseases such as higher risk of coronary heart disease, heart failure (5) and osteoporosis (6). Likewise, shorter telomeres were observed among individuals diagnosed with metabolic diseases such as obesity and type 2 diabetes mellitus, when compared to control subjects (7).

Although still disputed, available evidence suggests that lifestyle and dietary factors may affect telomere length (8). For instance, alcohol consumption and smoking were correlated with shorter telomere length in a dose-dependent fashion (9), while the consumption of antioxidant-rich foods such as vegetables, whole grains and fish was linked with longer telomeres (10). Moreover, higher adherence to the Mediterranean dietary pattern was linked to positive effects on telomere length, providing a greater insight on diet and its potential impact on the dynamics of telomere length (11). On the other hand, higher intake of sugar-sweetened beverages (SSB) was suggested to be linked with shorter telomere length (12, 13). It was argued that the link between SSB and telomere length may be explained by the sugar content of these beverages, and specifically fructose, given that high fructose intake may contribute to a biochemical environment that is characterized by oxidative stress and a high inflammatory response (12). However, none of the existing studies have examined the specific link between fructose intake and telomere length. It would be therefore important to further understand this link given it public health implications. In fact, fructose, a natural sugar present in fruits, is also increasingly used by the food industry given its high sweetness properties (14). One of the major ways to consume fructose is via sucrose or high fructose corn syrup, a form of added sugar found in commercial soft drinks, juices, and baked goods (15). Consequently the increase in the population’s exposure to added fructose has been underlined as an issue of health concern (15). Unlike age, dietary intake (including fructose intake) is a modifiable risk factor that can be modulated to prevent or delay telomere shortening and other adverse metabolic effects. It is in this context that this exploratory study was undertaken with the objectives of assessing the intake of dietary fructose (total, added and natural) in a sample of Lebanese healthy adults and investigating the association of fructose intakes with relative telomere length (RTL) in the study sample.

## 2. Materials and methods

The current study’s data was based on the cross-sectional survey titled “Assessment of Bisphenol A (BPA) levels and their association with the health status among the Lebanese population” (16). The original study was conducted on a representative sample of Lebanese adults living in the greater area of Beirut, and it was carried out between March and May 2014. The survey and study protocol were approved by the Institutional Review Board of the American University of Beirut (Protocol number IM.HT.03). Before enrolling participants in the study, all participants signed an informed consent.

### 2.1. Participants

Participants were adults from the Greater Beirut area, who were recruited through a multistage probability sampling approach, whereby the various districts were the strata. Using a systematic random sampling approach, a sample of neighborhoods and then households were chosen randomly from every district. At the household level, the adult individual with the latest birth month was selected to be part of the study, if eligible. Participants who were mentally disabled, pregnant or on dialysis were excluded from the study. Also, individuals employed in plastic or other chemical industries were not included due to the fact that they may have been exposed to BPA (and hence will not meet the eligibility criteria of the original study which evaluated the population’s exposure to BPA).

For the purpose of the current study, participants were selected from the parent study (*n* = 501) based on the following criteria: (1) healthy, with no history of chronic disease and (2) having complete data pertinent to anthropometric measurements, biochemical assessment and dietary intake. Participants reporting implausible energy intakes (EI) were excluded (<500 or >6,000 kcal/day). In total, 282 participants aged ≥ 18 years were included in the present study.

### 2.2. Data collection

Participants were invited to visit the Department of Nutrition and Food Sciences at the American University of Beirut after an overnight fast. Data on sociodemographic, lifestyle, and dietary characteristics as well as medical history were collected using a questionnaire. Data collection also comprised anthropometric and biochemical assessment, as well as blood pressure (BP) measurements. Trained personnel collected the data and administered the study questionnaire in an interview setting.

### 2.3. Socio-demographic and lifestyle characteristics

The socio-demographic characteristics that were included in the current study comprised gender, age (in years), marital status (married vs. not married), level of educational, monthly household income (expressed in terms of US dollars), and crowding index. Monthly household income was categorized into <600$, 600$ ≤ income ≤ 2,000$, and > 2,000$. Educational level was divided into “no schooling or primary school,” “intermediate school,” “secondary school or technical diploma,” and “university degree” (17, 18). Lifestyle characteristics included smoking status (i.e., non-smokers, current smokers of cigarette or nargile or ex-smokers), alcohol consumption (ever vs. never), coffee consumption (ever vs. never) as well as the hours of sleep per night during weekdays and weekends and physical activity level. Sleep habits were assessed using the Berlin questionnaire (19). Physical activity was evaluated with the short version of the international physical activity questionnaire (20) and was classified into 3 levels: low, moderate, and high.

### 2.4. Anthropometric, blood pressure, and biochemical measurements

Body weight was measured to the nearest 0.1 Kg using a calibrated electronic weighing scale (Inbody 3.0, Biospace Co., Ltd., Korea) while the subjects were wearing light clothes without shoes. A portable stadiometer (Seca 213, Germany) was used to measure height and it was recorded to the nearest 0.5 cm. The candidates were required to be in a standing position, flat against the measuring board and without shoes. Body mass index (BMI) was calculated as weight (Kg) divided by square of the height (meters) (21). Waist circumference (WC) was measured at the umbilical level, using a non-stretchable tape meter (Seca 26201, Germany), to the nearest 0.5 cm. Percent body fat was estimated using the Bioelectrical Impedance Analysis technique (Inbody 3.0, Biospace Co., Ltd., Alpha-Tec s.a.r.l.).

After a ten-minute rest, sitting BP was measured using a standard digital sphygmomanometer. BP measurements were collected twice and the mean of the two readings was recorded. Following the withdrawal of blood, fasting blood glucose (FBG) levels were analyzed using an enzymatic technique (Cobas 6000, Roche, Indianapolis, IN, USA) (17, 18). Serum triglycerides (TG), high-density lipoprotein cholesterol (HDL-C) and low-density lipoprotein cholesterol (LDL-C) levels were assessed via an enzymatic spectrophotometric method based on the Vitros 350 analyzer (Ortho-Clinical Diagnostics, Johnson and Johnson, 50–100 Holmers Farm Way, High Wycombe, Buckinghamshire, HP12 4DP, United Kingdom) (17, 18).

Metabolic syndrome (MetS) was identified according to the International Diabetes Federation (IDF) harmonized definition. Participants were categorized as having the MetS if they exhibited a minimum of 3 out of the following 5 metabolic anomalies: (1) Abdominal obesity: WC ≥ 80 cm for women and WC ≥ 94 cm for men (Eastern Mediterranean and Middle Eastern populations are encouraged to adopt the European cutoffs), (2) high BP: ≥130 mmHg systolic or ≥85 mmHg diastolic, (3) high fasting blood sugar: ≥100 mg/dL (5.6 mmol/L), (4) high TG levels: ≥150 mg/dL (>1.69 mmol/L), (5) low serum HDL-C levels: <50 mg/dL (<1.29 mmol/L) for women (22) and <40 mg/dL (<1.04 mmol/L) for men (17, 18).

### 2.5. Measurement of relative telomere length

Total deoxyribonucleic acid (DNA) was extracted from leucocytes of peripheral venous blood, through the use of the Qiagen kit (Qiagen, USA), based on the manufacturer’s instructions, whereby “it was normalized to a concentration of 10 ng/ul and stored at −20°C” until further analysis. Then, RTL was assessed using quantitative real-time polymerase chain reaction (RT-qPCR) on the CFX384 Touch Real-Time PCR Detection System from BIO-RAD, following the procedure outlined by Cawthon’s (23), with some modifications introduced by Cawthon (24). The calculation of RTL was then performed using Pfaffl’s formula, which accounts for varying plate efficiencies (25).

### 2.6. Dietary intake assessment

A semi-quantitative, culture specific food frequency questionnaire (FFQ) with 82-food items was utilized to collect dietary data, in an interview setting. The FFQ was designed to assess the participants’ usual dietary intake in the previous year. A reference portion size (shown in commonly used household measures such as spoons, cups, bowls, plates or common packing size) was provided for each of the food items included in the FFQ. To help the individual in determining the portion size, the standard two-dimensional food portion visual chart, developed by Nutrition Consulting Enterprises, was also used (26).

A database application was created using Microsoft Access (Microsoft Corp., Redmond, WA, USA) for data entry purposes. As such, the reported frequency of consumption for each beverage or food was translated into daily intake for further analysis. The Nutritionist Pro software, version 1.2 was used to calculate total energy and macronutrients intakes. Energy (kcal) was computed per gram for each food item/beverage on the FFQ list. The participant’s daily EI was then computed by summing the respective products of the quantity consumed and the energy per gram value for each food item/beverage (27). The same procedure was used to calculate the daily intake of each macronutrient (28), as well as for total fructose.

### 2.7. Estimation of added and natural fructose dietary intakes

Data on added vs. natural fructose is not available in the Nutritionist Pro database. Hence, the intake of naturally-occurring fructose from fructose-containing foods such as fruits, vegetables and honey, was considered as “natural fructose” (17, 18). The intake of fructose from processed foods and beverages which contain added sugar was defined as “added fructose.” Recognizing that the most common forms of added sugar in food products are sucrose or high fructose corn syrup, 50% of added sugar in food commodities was considered as added fructose (17, 29). The intake of total fructose was computed by adding up the respective intakes of natural fructose and added fructose (17, 18).

### 2.8. Statistical analysis

Statistical analysis was conducted via the Stata software [StataCorp., (30). Stata: Release 16. Statistical Software. StataCorp. LLC]. RTL was categorized into tertiles: 1st tertile corresponding to <1.12, 2nd tertile being between 1.12 and 1.55, and the 3rd tertile corresponding to >1.55.

Sociodemographic, lifestyle, anthropometric and biochemical characteristics, as well as dietary intakes of the total study sample and across RTL tertiles were computed as means with standard deviations (SD) for continuous variables, and as numbers and frequencies for categorical ones. The associations of potential predictors with RTL tertiles were initially assessed using one-way analysis of variance (ANOVA) or Chi-square test, where applicable. Multinomial logistic regression analysis was then performed to examine the associations between RTL tertiles (the multinomial outcome) and fructose intakes (main explanatory variable). Three models were adopted: model 1 for analyzing the association between tertiles of total fructose intake (in grams per day) and RTL tertiles; model 2 for analyzing the association between tertiles of added fructose intake (in grams per day) and RTL tertiles, and model 3 for analyzing the association between tertiles of natural fructose intake (in grams per day) and RTL tertiles. In all models, the highest tertile for RTL was used as the reference while the first tertile of fructose intake was taken as the reference. The selection of potential confounders that need to be adjusted for in these models was guided by existent literature (7, 31–38) and performed through the use of a directed acyclic graph (DAG), which was built using the DAGitty web application (39, 40). Accordingly, the minimal set of confounders selected for investigating the association between fructose intake and RTL included age, gender, income level, and education level (Appendix 1). Results stemming from the multinomial logistic analyses were reported as Relative Risk Ratio (RRR) with 95% confidence interval (CI). A *p*-value < 0.05 was set as indicator for statistical significance. It is important to note that no power analysis was conducted for this study because there is no literature on dietary fructose intakes in relation to RTL, in addition to the fact that this study used an already existing sample. Thus, this work is to be considered as exploratory and hypothesis generating.

## 3. Results

### 3.1. Characteristics of the study sample according to RTL tertiles

The socio-demographic and lifestyle characteristics of the study sample, according to RTL tertiles are shown in Tables 1, 2, respectively. Mean age of the study participants was of 41.01 ± 13.74 years, with a higher proportion of females compared to males (67.4 vs. 32.6%). Only (9.1%) of the study subjects had a monthly income greater than 2,000$ per month. More than 50% of the participants had up to intermediate educational level, with only 13.9% having accomplished university level. Results showed a significant association between older age and shorter RTL (*p* = 0.04) (Table 1).

**TABLE 1:** Sociodemographic characteristics of the study sample (*n* = 282) across RTL tertiles.

		RTL tertiles			
Sociodemographic characteristics	Total sample (*n* = 282)	< 1.12 (*n* = 94)	1.12–1.55 (*n* = 94)	>1.55 (*n* = 94)	*p*-value
	Mean ± SD				
	*n* (%)				
**Age (years)**					
<40	138 (48.9)	36 (38.3)	45 (47.9)	57 (60.6)	**0.04**
Between 40 and 60	119 (42.2)	48 (51.1)	41 (43.6)	30 (31.9)	
>60	25 (8.9)	10 (10.6)	8 (8.5)	7 (7.4)	
**Gender**					
Male	92 (32.6)	31 (33.0)	28 (29.8)	33 (35.1)	0.73
Female	190 (67.4)	63 (67.0)	66 (70.2)	61 (64.9)	
**Marital status**					
Married	192 (68.1)	63 (67.0)	63 (67.0)	66 (70.2)	0.86
Not married	90 (31.9)	31 (33.0)	31 (33.0)	28 (29.8)	
**Educational level**					
No school/primary	89 (31.7)	37 (39.4)	29 (31.2)	23 (24.5)	0.19
Intermediate	76 (27.0)	18 (19.1)	28 (30.1)	30 (31.9)	
Secondary/technical	77 (27.4)	24 (25.5)	27 (29.0)	26 (27.7)	
University	39 (13.9)	15 (16.0)	9 (9.7)	15 (16.0)	
**Income level**					
<600$	75 (28.5)	23 (27.1)	29 (33.3)	23 (25.3)	0.11
Between 600 and 2,000$	164 (62.4)	49 (57.6)	54 (62.1)	61 (67.0)	
>2,000$	24 (9.1)	13 (15.3)	4 (4.6)	7 (7.7)	
**Crowding index**					
≤1 person/room	109 (38.7)	38 (40.4)	35 (37.2)	36 (38.3)	0.90
>1 person/room	173 (61.3)	56 (59.6)	59 (62.8)	58 (61.7)	

RTL, relative telomere length; SD, standard deviation. Bold values indicate significance at *p* < 0.05.

**TABLE 2:** Lifestyle characteristics of the study sample (*n* = 282) across RTL tertiles.

		RTL tertiles			
Lifestyle characteristics	Total sample (*n* = 282)	< 1.12 (*n* = 94)	1.12–1.55 (*n* = 94)	> 1.55 (*n* = 94)	*p*-value^1^
	*n* (%)				
**Smoking status**					
Non-smoker	63 (22.3)	17 (18.1)	19 (20.2)	27 (28.7)	0.15
Current smoker (cigarette or nargileh)	194 (68.8)	68 (72.3)	70 (74.5)	56 (59.6)	
Ex-smoker	25 (8.9)	9 (9.6)	5 (5.3)	11 (11.7)	
**Alcohol consumption**					
Yes	47 (16.7)	16 (17.0)	19 (20.2)	12 (12.8)	0.38
No	235 (83.3)	78 (83.0)	75 (79.8)	82 (87.2)	
**Coffee consumption**					
Yes	220 (78.0)	73 (77.7)	80 (85.1)	67 (71.3)	0.07
No	62 (22.0)	21 (22.3)	14 (14.9)	27 (28.7)	
**Physical activity**					
None	42 (14.9)	18 (19.1)	13 (13.8)	11 (11.7)	0.33
Any	240 (85.1)	76 (80.9)	81 (86.2)	83 (88.3)	
**Levels of physical activity**					
Low-intensity activity	131 (46.5)	45 (47.9)	47 (50.0)	39 (41.5)	0.71
Moderate-intensity activity	87 (30.9)	30 (31.9)	25 (26.6)	32 (34.0)	
High-intensity activity	64 (22.7)	19 (20.2)	22 (23.4)	23 (24.5)	
**Number of sleep hours on weekdays**					
4 h or less	31 (11.0)	10 (10.6)	14 (14.9)	7 (7.4)	0.26
5 to 6 h	72 (25.5)	32 (34.0)	20 (21.3)	20 (21.3)	
6 to 7 h	77 (27.3)	27 (28.7)	23 (24.5)	27 (28.7)	
7 to 8 h	59 (20.9)	12 (12.8)	23 (24.5)	24 (25.5)	
8 to 9 h	29 (10.3)	8 (8.5)	9 (9.6)	12 (12.8)	
9 h or more	14 (5.0)	5 (5.3)	5 (5.3)	4 (4.3)	
**Number of sleep hours on weekends**					
4 h or less	31 (11.0)	10 (10.6)	12 (12.8)	9 (9.6)	0.14
5 to 6 h	49 (17.4)	22 (23.4)	15 (16.0)	12 (12.8)	
6 to 7 h	62 (22.0)	23 (24.5)	22 (23.4)	17 (18.1)	
7 to 8 h	61 (21.6)	12 (12.8)	24 (25.5)	25 (26.6)	
8 to 9 h	39 (13.8)	10 (10.6)	11 (11.7)	18 (19.1)	
9 h or more	40 (14.2)	17 (18.1)	10 (10.6)	13 (13.8)	
**Sleeping difficulties**					
Never	70 (24.8)	21 (22.3)	21 (22.3)	28 (29.8)	0.28
Rarely/sometimes/frequently	90 (31.9)	34 (36.2)	25 (26.6)	31 (33.0)	
Almost always	122 (43.3)	39 (41.5)	48 (51.1)	35 (37.2)	

^1^Chi-squared was done for significance of categorical variables.

RTL, relative telomere length.

As for the lifestyle factors, most of the participants were current cigarette or nargileh smokers (68.8%), and almost half had a low level of physical activity (46.5%). Concerning alcohol consumption, the majority of the participants had never consumed alcohol (83.3%). More than half of the population reported sleeping less than 8 h on weekdays and weekends. No significant differences were found in lifestyle characteristics across RTL tertiles (Table 2).

**FIGURE 1:**
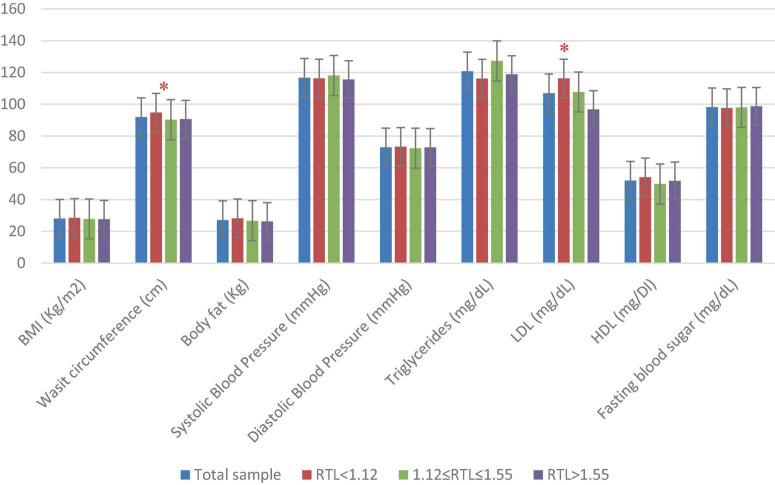
Anthropometric, blood pressure, and biochemical characteristics of the study population (*n* = 282) across RTL tertiles. One-way ANOVA test was done to compare for significance of continuous variables. BMI, body mass index; HDL, high-density cholesterol; LDL, low-density cholesterol; RTL, relative telomere length. Significant differences between the RTL tertiles are indicated as * for *p* < 0.05.

Figure 1 presents the sample’s anthropometric and biochemical measurements as well as BP data across RTL tertiles. The results showed that mean WC was significantly higher in participants with shorter RTL (*p* = 0.04). Similarly, mean LDL-C was significantly higher in participants with shorter RTL (*p* = 0.001).

### 3.2. Dietary intakes of the study participants according to RTL tertiles

Table 3 presents the dietary intakes of the study participants across RTL tertiles. The average intake of total dietary fructose was of 51.31 ± 35.55 g/day which represents 6.58 ± 3.71% of the total EI. Intakes of natural and added fructose were estimated at 12.28 ± 8.59 g/day and 39.03 ± 34.12 g/day (1.78 ± 1.41% and 4.80 ± 3.56% EI), respectively. Intakes of total sugar and total fructose were found to differ significantly across RTL tertiles (*p* = 0.02 and *p* = 0.03, respectively).

**TABLE 3:** Intakes of energy, macronutrients and fructose in the study sample (*n* = 282) across RTL tertiles.

			RTL tertiles			
Dietary intakes	Total sample (*n* = 282)		< 1.12 (*n* = 94)	1.12–1.55 (*n* = 94)	>1.55 (*n* = 94)	*p*-value^1^
	Mean ± SD	Median (IQR)	Mean ± SD			
**Energy and macronutrients intakes**						
Energy (kcal)	3,123 ± 1,290	2,913 (1,756)	3,015 ± 1,229	3,223 ± 1,203	3,131 ± 1,431	0.54
Carbohydrates (g)	387.38 ± 157.81	364.09 (210.20)	373.49 ± 150.25	403.23 ± 156.30	385.40 ± 166.70	0.43
Carbohydrates (% EI)	50.41 ± 8.29	50.76 (10.30)	50.37 ± 8.23	50.35 ± 8.01	50.51 ± 8.72	0.99
Protein (g)	102.19 ± 59.79	91.68 (62.00)	97.38 ± 44.32	102.04 ± 48.69	107.16 ± 80.11	0.53
Protein (% EI)	13.01 ± 3.64	12.37 (3.52)	12.97 ± 3.39	12.53 ± 2.59	13.52 ± 4.62	0.17
Fat (g)	131.19 ± 64.25	119.20 (88.40)	126.68 ± 64.76	136.08 ± 57.25	130.82 ± 70.40	0.60
Fat (% EI)	39.09 ± 7.87	38.36 (10.11)	38.87 ± 7.88	39.70 ± 8.34	38.69 ± 7.42	0.64
Saturated fat (g)	36.88 ± 20.23	32.59 (25.40)	36.36 ± 21.17	38.53 ± 18.73	35.76 ± 20.82	0.61
Saturated fat (% EI)	10.31 ± 2.72	10.21 (3.37)	10.34 ± 2.59	10.49 ± 2.70	10.11 ± 2.90	0.64
Fiber (g)	28.08 ± 11.72	26.27 (14.56)	27.16 ± 12.57	29.70 ± 11.59	27.37 ± 10.89	0.25
Total sugars (g)	104.80 ± 58.47	91.42 (66.23)	98.11 ± 54.60	118.50 ± 65.93	97.82 ± 52.14	**0.02**
**Fructose intakes**						
Total fructose (g)	51.31 ± 35.55	41.96 (37.23)	48.06 ± 36.02	59.06 ± 38.33	46.82 ± 31.01	**0.03**
Total fructose (% EI)	6.58 ± 3.71	5.96 (4.13)	6.48 ± 3.98	7.23 ± 3.94	6.03 ± 3.08	0.08
Natural fructose (g)	12.28 ± 8.59	10.34 (10.12)	12.04 ± 8.21	13.25 ± 9.97	11.54 ± 7.37	0.37
Natural fructose (% EI)	1.78 ± 1.41	1.42 (1.48)	1.88 ± 1.63	1.80 ± 1.46	1.65 ± 1.10	0.53
Added fructose (g)	39.03 ± 34.12	28.34 (36.33)	36.01 ± 34.08	45.80 ± 37.80	35.27 ± 29.28	0.06
Added fructose (% EI)	4.80 ± 3.56	4.00 (3.75)	4.59 ± 3.63	5.43 ± 3.96	4.38 ± 2.97	0.10

^1^One-way ANOVA test was done to compare for significance of continuous variables.

Bold values indicate significance at *p* < 0.05.

EI, energy intake; IQR, interquartile range; RTL, relative telomere length; SD, standard deviation.

### 3.3. Association between fructose intakes and RTL in the study sample

Table 4 summarizes the results of the multinomial logistic regression analysis examining the association between each of total, added, and natural fructose intakes in grams/day as tertiles and RTL tertiles, after adjustment for confounders (identified using DAG), which included gender, age, educational and income levels. The regression analysis results showed that the relative risk of having a shorter RTL (having an RTL between 1.12 and 1.55 as compared to having an RTL > 1.55) was of 3.10 [95% CI: (1.38, 6.94), *p* = 0.006] when consuming the highest tertile of total fructose (>53.6 g) relative to lower consumption (<30.9 g/day). Similarly, the relative risk of having a shorter RTL (having an RTL between 1.12 and 1.55 as compared to having an RTL > 1.55) was of 2.33 [95% CI: (1.02, 5.36), *p* = 0.045] when consuming the highest tertile of added fructose (>40.3 g) relative to lower consumption (<19.4 g/day) (Table 4). There was no significant association between fructose intakes and having an RTL < 1.12 (i.e., tertile 1) as compared to RTL > 1.55 (i.e., tertile 3) (Appendix 2 presents a graphical abstract of the study).

**TABLE 4:** Multinomial logistic regression analysis for the associations between each of total, added and natural fructose intakes (in grams/day) as tertiles and RTL tertiles.^1^

		RTL tertiles				
		Tertile 1		Tertile 2		Tertile 3
		Tertile < 1.12		RTL: 1.12–1.55		RTL: > 1.55
		RRR (95% CI)	*p*-value	RRR (95% CI)	*p*-value	
Total fructose intake (g/day)	1^st^ tertile: < 30.9	Reference				
	2^nd^ tertile: 30.9–53.6	0.90 (0.43, 1.88)	0.784	1.24 (0.57, 2.72)	0.584	Reference
	3^rd^ tertile: > 53.6	1.07 (0.47, 2.43)	0.876	**3.10 (1.38, 6.94)**	**0.006**	Reference
Added fructose intake (g/day)	1^st^ tertile: < 19.4	Reference				
	2^nd^ tertile: 19.4–40.3	0.74 (0.35, 1.57)	0.436	0.98 (0.45, 2.12)	0.950	Reference
	3^rd^ tertile: > 40.3	1.12 (0.48, 2.62)	0.793	**2.33 (1.02, 5.36)**	**0.045**	Reference
Natural fructose intake (g/day)	1^st^ tertile: < 7.7	Reference				
	2^nd^ tertile: 7.7–13.8	1.33 (0.61, 2.89)	0.466	0.85 (0.40, 1.81)	0.682	Reference
	3^rd^ tertile: > 13.8	0.99 (0.46, 2.19)	0.999	0.98 (0.47, 2.05)	0.963	Reference

^1^The three models were adjusted for age, gender, education level, and income level.

Bold values indicate significance at *p* < 0.05.

CI, confidence interval; RRR, relative risk ratio; RTL, relative telomere length.

## 4. Discussion

This exploratory study is the first to investigate the association between dietary fructose intake and telomeres’ length. In a cross-sectional design, the study findings suggest a potential association between higher intakes of total/added fructose and shorter RTL in a sample of Lebanese urban adults, while no association was found with natural fructose. The study also showed a significant association of shorter telomere length with higher age, elevated WC and higher LDL-levels.

The observed association between shorter telomeres’ length with age is in agreement with that reported by several previous studies where telomere shortening was described as gradual with aging and was linked to age-related diseases (31, 32, 35). Our study also showed a significant association between increased WC and shorter telomere length. These findings are in line with those reported by a previous study conducted in 2018 amongst 7,827 subjects, based on the National Health and Nutrition Examination Survey (NHANES) 1999–2002 (41), and those observed amongst Chinese women (*n* = 2,912) aged 40–70 years old, where an inverse association between WC and telomere length was reported (42). A significant relationship was also found between LDL-C and RTL tertiles in our study. Consistent with these findings, a study on 4,944 subjects from the population-based Gutenberg Health Study showed an association between telomere length and higher LDL-C levels in the oldest tertile of age (33). Another study that examined 305 subjects in Belgium also showed that age and gender-adjusted telomere length was inversely associated with LDL-C (34). Other studies, however, did not find such associations (43).

Given that available evidence suggests that lifestyle and dietary factors may affect telomere length (8), the present study aimed at investigating the association of dietary intakes, and particularly fructose intake, with telomere length. The study showed that total dietary fructose intake was of 51.31 ± 35.55 g/day, a value that slightly exceeds the proposed upper limit for fructose intake (>50 g/day) (18, 44). In addition to the estimation of total fructose intake, we have also performed an assessment of added and natural fructose intakes given that natural fructose, which is present in fruits and vegetables, is not hypothesized to contribute to telomere shortening (45). In fact, fruits and vegetables include a wide range of nutrients, phytochemical and antioxidants that may actually prevent or delay telomere shortening (46), In our study, the average intake of added fructose was of 39.03 ± 34.12 g/day, providing approximately 4.8% of EI, while that of natural fructose was of 12.3 g/day ± 8.59, contributing 1.8% of EI. Few studies have undertaken such evaluations, which limits our ability to compare our findings with the literature. A study conducted in Iran estimated added fructose intake at 26.9 ± 13.9 and 19 ± 13.7 g/day in men and women, respectively (17), estimates that are lower than those obtained in the current study.

In line with our hypothesis, the study findings did not show any association between natural fructose intake and RTL. However, although we could not observe a dose-dependent relation between fructose intakes and RTL shortening, the study results showed a significant association between higher intakes of total fructose (tertile 3) with shorter RTL (i.e., having an RTL between 1.12 and 1.55 as compared to having an RTL > 1.55). A similar association was observed between higher intakes of added fructose and shorter RTL. These findings are in line with those reported by studies focusing on SSB, which is a significant contributor to added fructose intake, and which showed that higher consumption of SSB was linked with shorter telomere length (12, 13). Similarly, our observations are in line with those described by previous narrative reviews, where a correlation between diet and telomeres’ shortening was reported (47). In a cross-sectional study that examined associations between SSBs and telomere length in a nationally representative sample of healthy adults from the NHANES (*N* = 5,309), Leung et al. (13) found that the consumption of sugar-sweetened soda was linked to shorter telomeres. In line with our hypothesis, Leung et al. (13) suggested that this association could be attributed to the impact of SSBs on oxidative stress, systemic inflammation, and insulin resistance (48, 49). According to Miller and Adeli (14), the production of advanced glycation end products (AGEs) by fructose is 10 folds higher than that of glucose, and high levels of AGEs can promote the production of reactive oxygen species (ROS) and hence mediate inflammatory pathways (50). Similarly, hyperlipidemia induced by excessive fructose consumption can lead to lipid retention in the hepatic cells, which in turn can trigger the release of ROS and activation of inflammatory cytokines at different intracellular levels (51). It is, however, important to acknowledge that other studies did not observe a relation between dietary exposure and RTL (52). For example, a cross-sectional study conducted among 840 white, black, and Hispanic adults from the Multi-Ethnic Study of Atherosclerosis, no associations were found between telomere length and sugar-sweetened soda consumption (53).

### 4.1. Strengths and limitations

The present study has several strengths. It was in fact conducted on a random sample of adults living in the Greater Beirut area and adopted a well-designed protocol and methodology. The dietary assessment conducted in this study allowed us to estimate the intakes of added vs. natural fructose, rather than total fructose alone. In addition, and in order to minimize potential reverse causation, participants who reported having chronic diseases or metabolic abnormalities that might have had an impact on their dietary patterns were excluded from the study.

However, this study has several limitations. First, the study findings are limited by its small sample size, as it builds on an already available sample. Therefore, this work is to be considered exploratory in nature and hypothesis generating. The study’s findings are also limited by its cross-sectional design which does not allow to assess causality. It was, in fact, not possible for us to take into consideration longitudinal changes in telomere length in relation to diet. Moreover, it may be possible to argue that although adjustment for demographic and lifestyle factors that were previously shown to be associated with telomere length was carried out, the presence of residual confounding could remain a possibility that might have affected our results. Although every effort was done to measure these covariates accurately, residual confounding could be unavoidable, to some degree (53). The study may also have sources of potential bias or imprecision. For instance, we have used a semi-quantitative FFQ for the collection of dietary data, which, similarly to other dietary assessment methods, may be associated with recall bias and misreporting of intakes (54). Although the FFQ has its limitations, it was found to be amongst the most appropriate tools for dietary assessment in large-scale epidemiological research, and particularly in the ranking of individuals based on their dietary intakes (55). The FFQ is also an appropriate method for the estimation of dietary intake over an extended period of time (56). The fact that data collection in our study was conducted in an interview setting may have led to a social desirability bias and misreporting of dietary intakes in a way that is perceived as favorable to the interviewer (57). Accordingly, this bias may have resulted in an underestimation of added fructose intakes and hence may have diluted the observed associations., In addition, in our study, data collection was performed by fieldworkers who had received extensive training to reduce judgmental verbal and non-verbal communication in order to minimize any social desirability bias. In our study, imprecisions may also have resulted from the food composition database that we have used for the assessment of fructose intake. In fact, in our study, food composition data were retrieved from the USDA database within the Nutritionist Pro software, given that specific food composition databases for Lebanon or the region are lacking. It is important therefore to acknowledge that the USDA database may not accurately capture the nutrient composition of all varieties of food and beverages available in Lebanon, which may constitute a source of error or imprecision in intake estimations (58). Moreover, the USDA database does not encompass several of the mixed traditional dishes/deserts consumed in the country. In order to tackle this limitation, we have created and added standardized recipes to the software using single food items (58). Techniques for measuring telomere length could also be a source of error or imprecision. In our study quantitative PCR (qPCR) technique was used, which is a technique that is widely used in DNA samples. However, it is challenging to optimize results, and variability has been reported (59). As imprecisions may dampen statistical associations, the results of this study on the link between fructose intakes and RTL may have in fact been weakened by the potential imprecisions described above. Finally, the present study was restricted to an urban setting, and thus, findings pertinent to the consumption levels of fructose may not be representative of less urban settings in the country. The choice of Beirut for this study may be explained by the fact that it hosts 40% of the Lebanese population and is usually considered a melting pot of the country (60).

## 5. Conclusion

In conclusion, although we could not observe a dose-dependent relation between fructose intakes and RTL shortening and although the study is limited by its small sample size, the findings suggest that total and added dietary fructose intakes were associated with shorter telomere length in a sample of Lebanese urban adults. These results therefore suggest a potential link between total and added fructose intakes and telomere length. Larger studies, of longitudinal nature, are needed to validate and better understand the association between fructose intakes and telomere length.

## Data availability statement

The raw data supporting the conclusions of this article will be made available by the authors, without undue reservation.

## Ethics statement

The studies involving humans were approved by the Institutional Review Board (American University of Beirut). The studies were conducted in accordance with the local legislation and institutional requirements. The participants provided their written informed consent to participate in this study.

## Author contributions

NM: Formal analysis, Writing – original draft. NZ: Data curation, Formal analysis, Writing – review and editing. FC: Formal analysis, Writing – review and editing. MN: Data curation, Writing – review and editing. HT: Data curation, Formal analysis, Resources, Writing – review and editing. LN: Conceptualization, Investigation, Supervision, Writing – review and editing.
