# Supplementary material for: Fructose intake and its association with relative telomere length: an exploratory study among healthy Lebanese adults
Source: Front Nutr. 2023 Oct 31;10:1270124. doi: 10.3389/fnut.2023.1270124 (PMC10643745; doi:10.3389/fnut.2023.1270124)
Supplement: Supplementary file 1 [file Presentation_1.PPTX]

## Slide 1
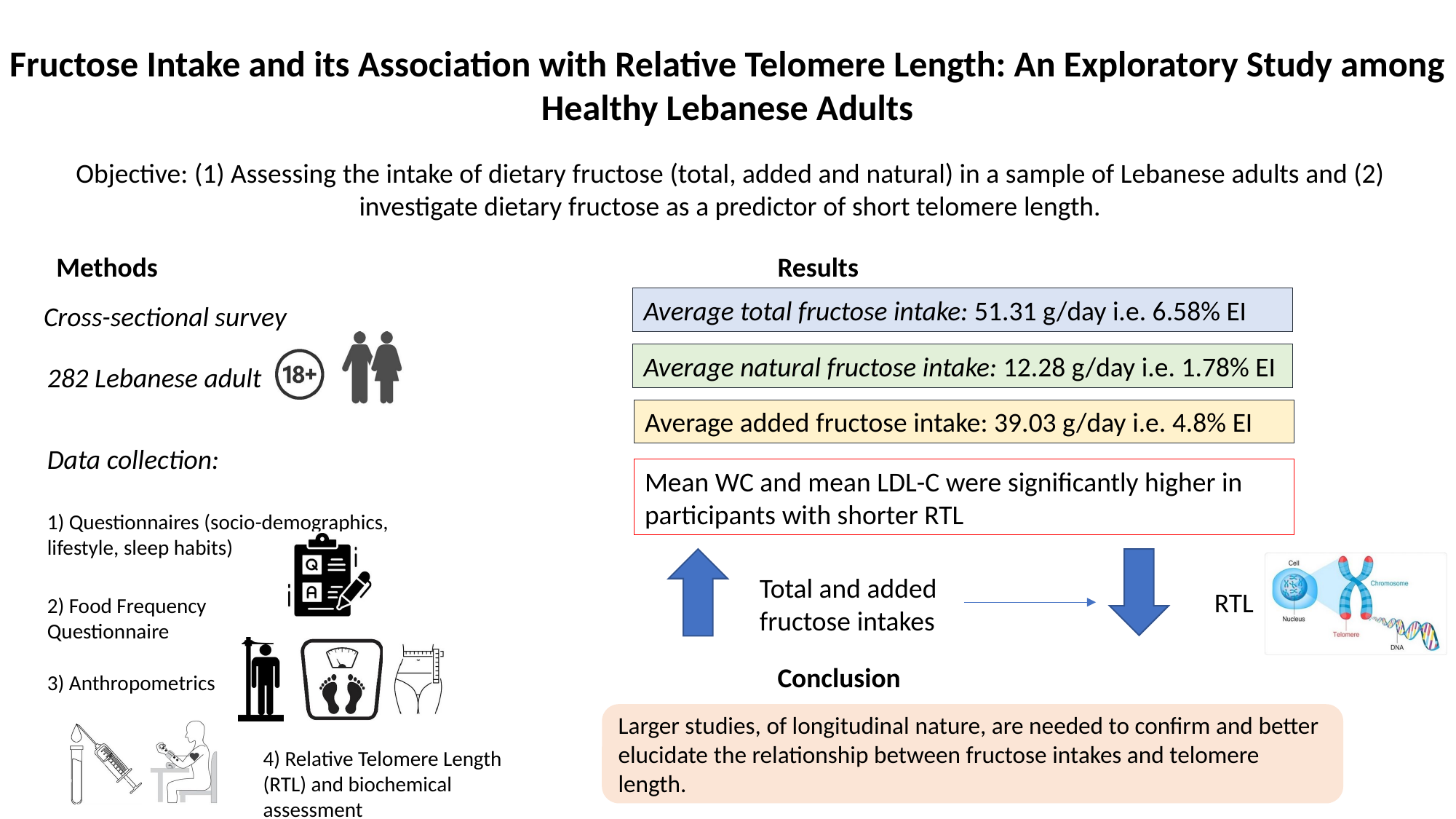

Fructose Intake and its Association with Relative Telomere Length: An Exploratory Study among Healthy Lebanese Adults
Objective: (1) Assessing the intake of dietary fructose (total, added and natural) in a sample of Lebanese adults and (2) investigate dietary fructose as a predictor of short telomere length.
Methods
Results
Average total fructose intake: 51.31 g/day i.e. 6.58% EI
Cross-sectional survey
Average natural fructose intake: 12.28 g/day i.e. 1.78% EI
282 Lebanese adult
Average added fructose intake: 39.03 g/day i.e. 4.8% EI
Data collection:
Mean WC and mean LDL-C were significantly higher in participants with shorter RTL
1) Questionnaires (socio-demographics, lifestyle, sleep habits)
Total and added fructose intakes
RTL
2) Food Frequency Questionnaire
Conclusion
3) Anthropometrics
Larger studies, of longitudinal nature, are needed to confirm and better elucidate the relationship between fructose intakes and telomere length.
4) Relative Telomere Length (RTL) and biochemical assessment
