# Supplementary material for: Fructose intake and its association with relative telomere length: an exploratory study among healthy Lebanese adults
Source: Front Nutr. 2023 Oct 31;10:1270124. doi: 10.3389/fnut.2023.1270124 (PMC10643745; doi:10.3389/fnut.2023.1270124)
Supplement: Supplementary file 2 [file Data_Sheet_1.docx]

**Appendix 1: Theoratical model of the association between fructose intake and Relative Telomere Length (RTL) based on a directed acyclic graph (DAG).**


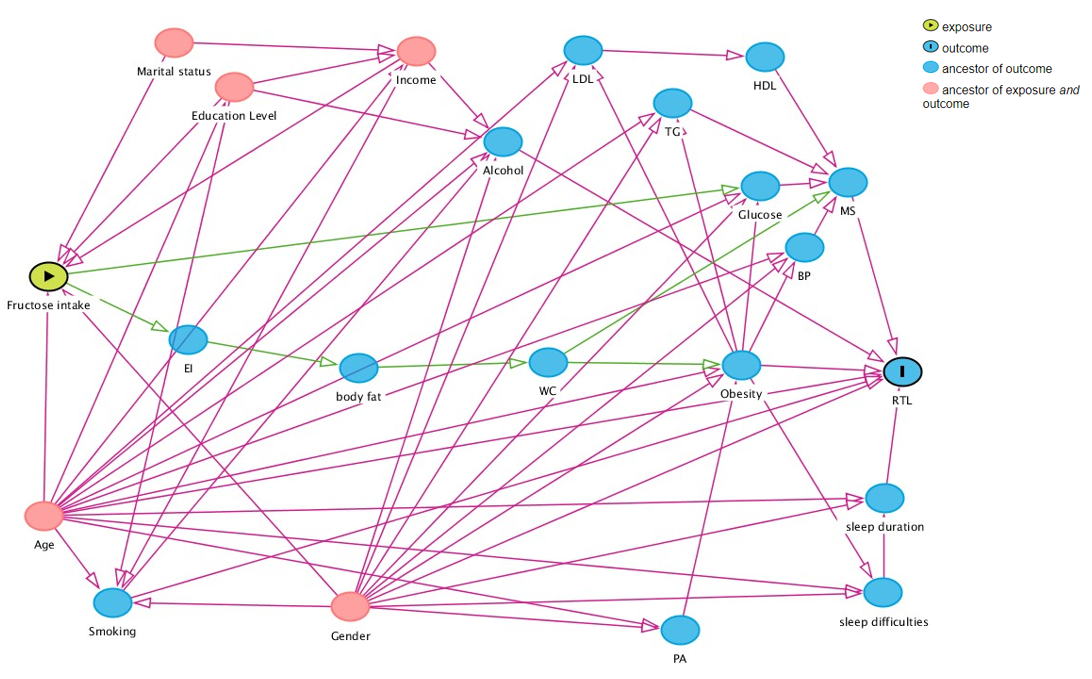


Nodes represent variables and arrows represent causal associations. Fructose intake is the exposure and RTL is the outcome. Abbreviations: EI: Energy Intake; PA: Physical Activity; TG: Triglycerides; WC: Waist Circumference; BP: Blood Pressure; MS: Metabolic Syndrome; LDL: Low Density Lipoprotein; HDL: High Density Lipoprotein; RTL: Relative Telomere Length. Minimal sufficient adjustment set for estimating the total effect of fructose intake on RTL include: age, gender, education level, and income level.
